# Aberrantly High Levels of Somatic LINE-1 Expression and Retrotransposition in Human Neurological Disorders

**DOI:** 10.3389/fgene.2019.01244

**Published:** 2020-01-08

**Authors:** Diane M. Terry, Scott E. Devine

**Affiliations:** ^1^Molecular Medicine Graduate Program, University of Maryland School of Medicine, Baltimore, MD, United States; ^2^Institute for Genome Sciences, University of Maryland School of Medicine, Baltimore, MD, United States; ^3^Department of Medicine, University of Maryland School of Medicine, Baltimore, MD, United States; ^4^Greenebaum Comprehensive Cancer Center, University of Maryland School of Medicine, Baltimore, MD, United States

**Keywords:** brain, retrotransposition, LINE, L1, SINE, somatic, neurological disease

## Abstract

Retrotransposable elements (RTEs) have actively multiplied over the past 80 million years of primate evolution, and as a consequence, such elements collectively occupy ∼ 40% of the human genome. As RTE activity can have detrimental effects on the human genome and transcriptome, silencing mechanisms have evolved to restrict retrotransposition. The brain is the only known somatic tissue where RTEs are de-repressed throughout the life of a healthy human and each neuron in specific brain regions accumulates up to ∼13.7 new somatic L1 insertions (and perhaps more). However, even higher levels of somatic RTE expression and retrotransposition have been found in a number of human neurological disorders. This review is focused on how RTE expression and retrotransposition in neuronal tissues might contribute to the initiation and progression of these disorders. These disorders are discussed in three broad and sometimes overlapping categories: 1) disorders such as Rett syndrome, Aicardi-Goutières syndrome, and ataxia–telangiectasia, where expression/retrotransposition is increased due to mutations in genes that play a role in regulating RTEs in healthy cells, 2) disorders such as autism spectrum disorder, schizophrenia, and substance abuse disorders, which are thought to be caused by a combination of genetic and environmental stress factors, and 3) disorders associated with age, such as frontotemporal lobar degeneration (FTLD), amyotrophic lateral sclerosis (ALS), and normal aging, where there is a time-dependent accumulation of neurological degeneration, RTE copy number, and phenotypes. Research has revealed increased levels of RTE activity in many neurological disorders, but in most cases, a clear causal link between RTE activity and these disorders has not been well established. At the same time, even if increased RTE activity is a passenger and not a driver of disease, a detrimental effect is more likely than a beneficial one. Thus, a better understanding of the role of RTEs in neuronal tissues likely is an important part of understanding, preventing, and treating these disorders.

## Introduction

Approximately 45% of the human genome is derived from insertions of DNA sequences known as transposable elements (TEs) (Lander et al., 2001). There are two types of transposable elements: DNA transposons and RNA transposons (aka, retrotransposable elements, or RTEs). DNA transposons, which use cut-and-paste or replicative mechanisms to move DNA copies from one place to another (McClintock, 1950), make up only ∼3% of the human genome (Lander et al., 2001). Although DNA transposons were active during early primate evolution, it is widely believed that they are no longer active in humans due to disabling mutations that have led to their extinction (Pace and Feschotte, 2007). In contrast, RTEs are still active and have served as a novel source of genetic diversity over the past 80 million years of primate evolution (Brouha et al., 2003; Beck et al., 2011). RTEs are classified as long terminal repeat (LTR) or non-LTR RTEs based on whether they have LTRs. Human endogenous retroviruses (HERVs) are LTR retrotransposons that make up ∼8% of the human genome (Lander et al., 2001). It is likely that most, if not all HERVs are no longer able to retrotranspose due to the accumulation of detrimental mutations in HERV copies. In contrast, non-LTR RTEs, which are the focus of this review, make up ∼34% of the human genome (Lander et al., 2001) and continue to actively create genetic diversity, mutations, and disease.

There are two types of non-LTR retrotransposons in humans: Long interspersed elements (LINEs), and short interspersed elements (SINEs) (Lander et al., 2001). Long interspersed element-1 (LINE-1, or L1) is the most successful transposable element family in humans and makes up ∼17% of the human genome (Lander et al., 2001). L1 elements are the only active autonomous retrotransposons in the human genome that encode the necessary “machinery” for their own mobilization. The two most common SINEs that are active in the human genome are *Alu* (∼11%) (Mills et al., 2007) and SINE-VNTR-*Alu* (SVA) (∼0.2%) (Mills et al., 2007). They are non-autonomous in that they do not encode their own mobilization machinery and instead efficiently hijack the L1 machinery to mobilize their own sequences (Mills et al., 2007; Cordaux and Batzer, 2009; Hancks et al., 2011; Raiz et al., 2012).

**Figure 1:**
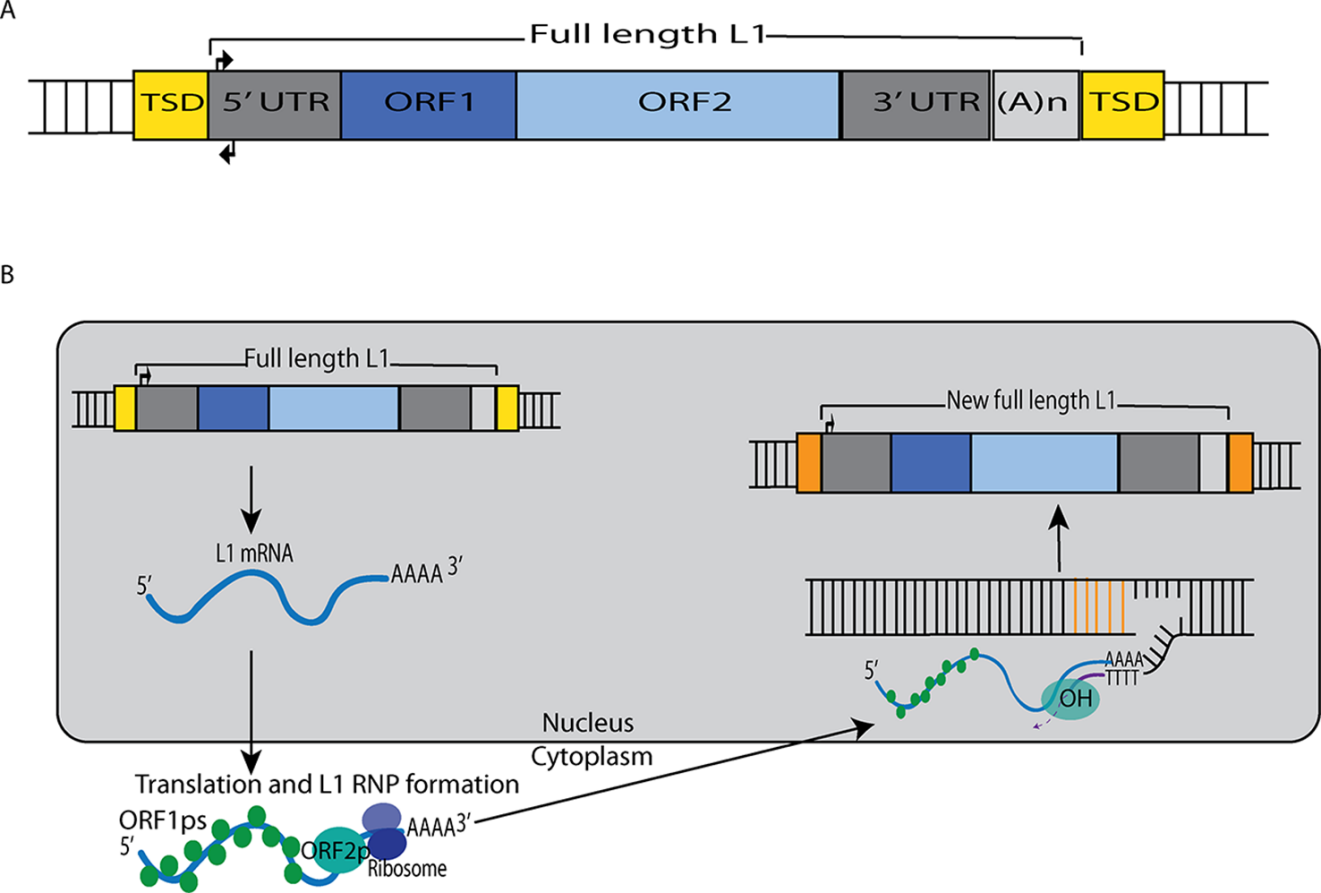
L1 retrotransposition mechanism. **(A)** A retrotransposition-competent L1 is 6 kb in length and consists of a 5′ UTR, open reading frame 1 (ORF1), open reading frame 2 (ORF2), a 3′ UTR and a poly(A) tail (Moran et al., 1996; Babushok and Kazazian, 2007; Rosser and An, 2012). Within the 5′UTR there is a promoter on the sense strand that drives the expression of full-length L1s as well as an antisense promoter. ORF1 codes for a protein (ORF1p) with RNA binding and nucleic acid chaperone activity, and ORF2 encodes for a protein (ORF2p) with endonuclease and reverse transcriptase activities. **(B)** After transcription, the L1 mRNA moves to the cytoplasm where it is translated. The resulting ORF1p and ORF2p preferentially bind to the L1 mRNA that produced them (cis-preference), forming an L1-RNP that is imported back to the nucleus. Insertion occurs through target primed reverse transcription in which the ORF2p endonuclease nicks a DNA strand at a 5′TTTT/AA3′ consensus site, thereby exposing a 3′ hydroxyl that serves as a priming site for the ORF2p reverse transcriptase to generate a cDNA copy from the L1 mRNA. It is believed that a similar process occurs on the other strand of DNA to complete the L1 insertion process. Only a fraction of the L1 insertions in humans are full-length (6 kb), as new insertions often are 5′ truncated due to DNA repair pathways that recognize and disrupt reverse transcription (Coufal et al., 2011).

Although the reference human genome contains ∼500,000 copies of L1s, only about 80–100 of these copies are full-length and encode the two open reading frames (ORF1 and ORF2) that are necessary for retrotransposition (**Figure 1**). Forty of these full-length elements were shown to be active in a cell-culture-based assay for retrotransposition, and six were highly active in this assay (Brouha et al., 2003; Beck et al., 2011). Additional retrotransposition-competent L1s have been identified in eight diverse (non-reference) humans, and many of these elements also were highly active in the cell culture assay (Beck et al., 2010). Full-length L1s with these properties produce new “offspring” L1 insertions elsewhere in the genome through a cycle of retrotransposition that is outlined in **Figure 1**. *Alu*s (Weichenrieder et al., 2000) and SVAs (Hancks et al., 2011) are mobilized in trans by hijacking the L1 machinery during this cycle, likely by docking their RNAs on ribosomes and “stealing” the ORF1p and ORF2p proteins as they are being synthesized from L1 mRNAs (Boeke, 1997; Mills et al., 2007).

## L1 Activity in Somatic Cells of the Healthy Human Brain

Although *de novo* L1 insertions were initially thought to occur only in the germline, it is now clear that somatic L1 retrotransposition occurs in the healthy human brain in neural progenitor cells (NPCs) and during neurogenesis (Muotri et al., 2005; Coufal et al., 2009; Baillie et al., 2011; Evrony et al., 2012; Evrony et al., 2015; Upton et al., 2015). The first demonstration of L1 retrotransposition in a neuronal lineage was reported by Muotri et al. using a plasmid-based assay for L1 retrotransposition (Muotri et al., 2005). A plasmid carrying a human L1 element with a retrotransposition indicator cassette was introduced into a variety of neuronal and non-neuronal cells, including: rat adult hippocampus-derived neural progenitor (AHNP) cells, rat hippocampus neural stem (HCN) cells, rat primary neurons and astrocytes derived from the hippocampus, rat mesenchymal stem cells, rat ﬁbroblasts, and human CD34+ lymphocytes. Human HeLa cells, which are known to support L1 retrotransposition in this plasmid-based assay (Moran et al., 1996), also were included as a positive control for these experiments. L1 retrotransposition only occurred in AHNP, HCN, and HeLa cells in these experiments (Muotri et al., 2005).

**Figure 2:**
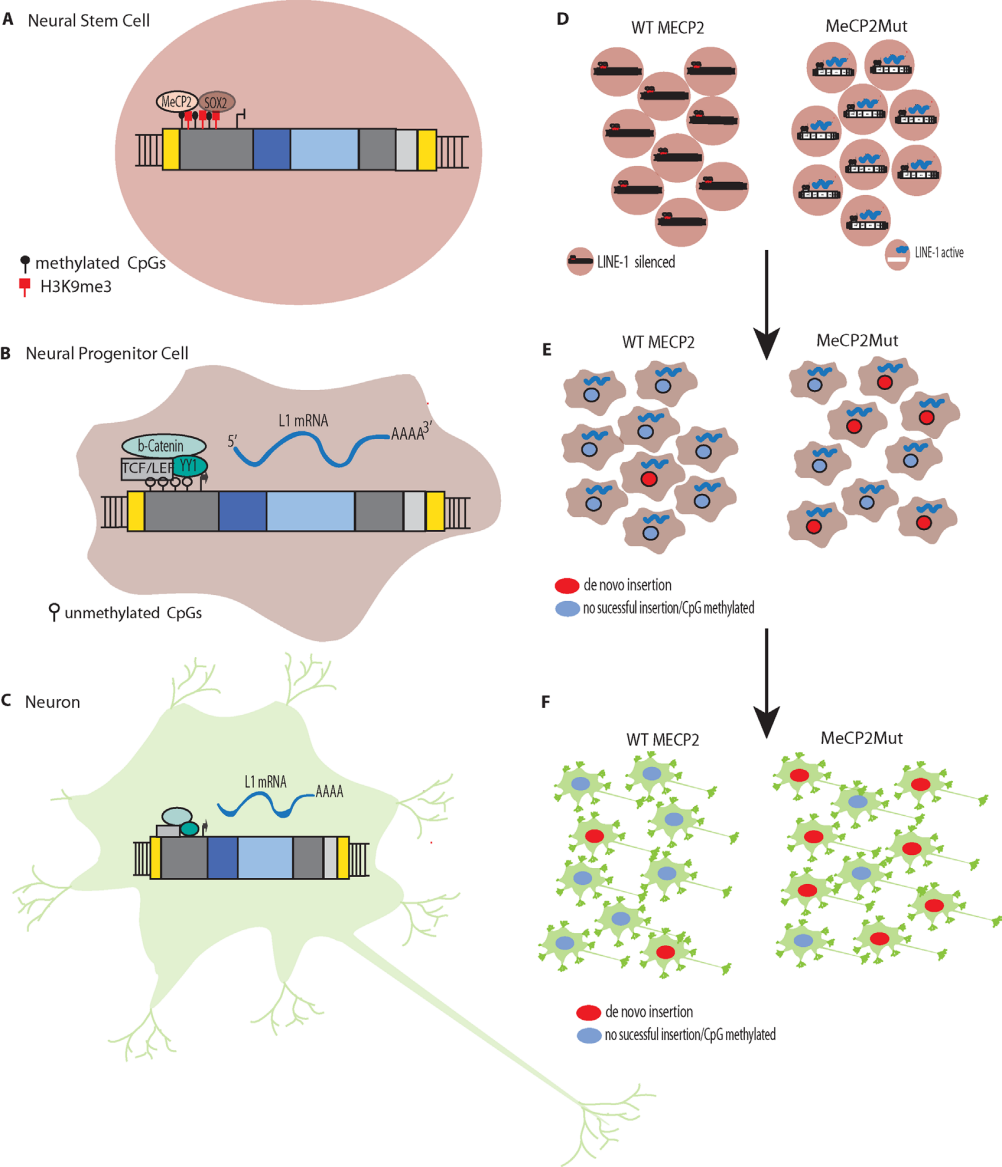
L1 Silencing **(A)** Methylation of CpG islands in the promoters of L1s to silence expression. L1 silencing is maintained by MeCP2p binding to methylated cytosines in the CpG island core of the L1 promoter. **(B)** The transcription factor SOX2 is downregulated during neural stem cell differentiation. The SOX2 downregulation, combined with chromatin remodeling and promoter demethylation, decrease MeCP2p binding to the L1 promoter. Simultaneous activation of the canonical WNT pathway results in stimulation of L1 expression. L1 *de novo* retrotransposition then can occur in the healthy human brain in neural progenitor cells (NPCs) and during neurogenesis, including in adults. **(C)** Retrotransposition does not just occur during differentiation but can occur in mature non-dividing neuronal cells. **(D)** In Rett syndrome, mutation of the X-linked gene methyl CpG binding protein 2 (MECP2) leading to abnormal epigenetic regulation such that L1 promoters are not silenced. **(E)**. The reduced regulation of L1 results in more expression and increased retrotransposition in neural progenitor cells (NPCs). **(F)** The increased retrotransposition events can continue to occur in mature non-dividing neuronal cells.

Muotri et al. (2005) also found that, as NPCs differentiated, the L1 promoter became transcriptionally active in response to decreased levels of repressive Sox2p (**Figure 2**). Likewise, they demonstrated that new L1 insertions could alter neuronal gene expression and inﬂuence neuronal cell fates *in vitro*. To validate the retrotransposition results *in vivo*, they developed transgenic mice harboring a retrotransposition–competent human L1 element under the control of its endogenous promoter. With this mouse model, they found mosaic L1 insertions in neuronal tissues but not in other somatic tissues. Overall, these experiments persuasively demonstrated that L1 can generate somatic L1 insertions in neuronal progenitor and neural stem cells (and likely in differentiating neurons).

Coufal et al. (2009) demonstrated that human NPCs isolated from human fetal brain stem cells and human embryonic stem cells (hESCs) also support L1 retrotransposition. They provided further evidence for L1-mediated somatic mosaicism in the brain using ex vivo quantitative PCR (qPCR) to measure endogenous L1 ORF2 copy number in the postmortem hippocampus, cerebellum, liver and heart from three healthy adult humans. Signiﬁcantly higher L1 copy numbers were observed in various regions of healthy human adult brains (particularly the hippocampus) compared to the livers and hearts of the same individuals. They estimated a theoretical increase in L1 ORF2 copy number of approximately 80 copies per cell based on samples from the hippocampus (Coufal et al., 2009). It is unclear whether the L1 copy number estimates that are measured by qPCR in these experiments are detecting new retrotransposition events in the genomes of these tissues, extrachromosomal intermediates, or perhaps both. Caution should be used here and in the remainder of this review in interpreting qPCR-based L1 copy number data.

Baillie et al. (2011) developed a high-throughput protocol called retrotransposon-capture sequencing (RC-seq) to map somatic retrotransposition events in the postmortem hippocampi and caudate nuclei of three healthy individuals. These two tissues were selected because the hippocampus had the highest L1 copy number, and the caudate nucleus had the lowest L1 copy number, in preliminary experiments. Several thousand L1, *Alu* and SVA germline insertions were identified, along with ∼22,000 somatic insertions (7,743 L1s, 13,692 *Alu*s, and 1,350 SVAs). They selected 33 candidates for validation experiments (14 full-length L1s, 15 full-length *Alu*s, and 4 SVAs) and were able to verify 28/33 (85%) by PCR. They found that in contrast to germline insertions, somatic insertions were disproportionately located in genes associated with neurogenesis and synaptic function, suggesting that retrotransposition may alter the genetic circuitry of neurobiological processes (Baillie et al., 2011).

Single cell experiments also have revealed that somatic retrotransposition occurs in the brain, although there is some disagreement about the precise amount of somatic retrotransposition that occurs (Evrony et al., 2012; Upton et al., 2015; Erwin et al., 2016; Sanchez-Luque et al., 2019). In one study, where genome-wide sequencing was used to detect full-length L1 insertions in 300 single neurons originating from the postmortem cerebral cortexes and caudate nuclei of three healthy individuals, Evrony et al. (2012) estimated ∼1.1 somatic insertions per neuron and <0.6 unique somatic insertions per neuron. Likewise, Erwin et al. estimated that somatic L1 insertions occur at a rate of 0.58–1 events per cell in both glia and neurons and affect ∼44–63% of the cells in the healthy brain (Erwin et al., 2016). In contrast, Upton et al. estimated a minimum of 13.7 somatic L1 insertions per hippocampal neuron and 6.5 insertions per glial cell, based on RC-seq measurements of postmortem samples from four individuals (Upton et al., 2015). The differences in these studies could be due to several factors, including: i) different brain tissues were examined, ii) the sample sizes of the studies were small (3–4 postmortem brain samples each), iii) different methods were used, and/or iv) the somatic RTE generation rates might have varied for the individuals studied (perhaps due to differences in L1 source element profiles).

Macia et al. (2017) recently found that there is efﬁcient L1 retrotransposition in nondividing mature neuronal cells, which comprise the majority of the cells in the human brain. A range of human cell types were differentiated from the same hESC line (H9) and tested for their ability to support L1 retrotransposition. Each differentiated cell type was infected with an adenovirus–retrotransposon hybrid virus containing a human L1 element with an EGFP reporter cassette. The study initially found that somatic expression and retrotransposition of L1s is restricted to a minor population of neuronal cells with multipotent characteristics. However, additional experimentation revealed that, in mature neuronal cells, the amount of L1–EGFP copies was approximately six-fold higher than in multipotent NPCs. These results initially were missed because the L1 constructs used in earlier experiments were not ideal for detecting L1 activity in these tissues. However, these studies clearly demonstrated that mature neuronal cells support somatic L1 retrotransposition. Thus, taken together with the Muotri et al. and Coufal et al. studies outlined above, it appears that somatic L1 retrotransposition occurs in: 1) differentiating neural stem cells, 2) neuronal progenitor cells, 3) differentiating neurons, and 4) mature neurons (**Figure 2**).

## Somatic L1 Retrotransposition in Brain Disorders

Kazazian et al. (1988) were the first to demonstrate that germline L1 retrotransposition events could cause human diseases. In particular, two independent germline L1 insertions that disrupted different sites within exon 14 of the Factor XIII gene caused Hemophilia in two unrelated patients. Since then, ∼125 germline RTE insertions have been identified that have caused Mendelian diseases by disrupting genes (Hancks and Kazazian, 2016). Somatic L1 insertions (including mosaic L1 insertions generated in early development and others generated in specific adult somatic tissues) also contribute to human diseases (Brouha et al., 2002; van den Hurk et al., 2007; Evrony et al., 2015; Richardson et al., 2017; Scott and Devine, 2017). As discussed above, L1 has the ability to generate new somatic L1 insertions in the human brain and growing evidence suggests that the mis-regulation of L1 in brain tissues is associated with neurological disorders. These disorders fall into three broad and sometimes overlapping categories: 1) disorders where RTE expression/activity is increased due to mutations in genes, which in healthy cells, regulate RTEs, 2) disorders with both genetic and environmental components that influence RTEs, and 3) disorders associated with age, i.e., where there is a time-dependent accumulation of neuronal degeneration, L1 copy number, and phenotypes associated with aging. A summary of the disorders, causes, and effects with respect to L1 activity is provided in **Table 1**.

**Table 1:** L1 related disorders, causes, and effects.

Disorder	Causes	Effect with respect to L1 activity
Rett Syndrome	Mutation of the X-linked gene methyl CpG binding protein 2 (MECP2).	Abnormal epigenetic regulation of L1s and other genes resulting in increased L1 expression and retrotransposition.
Aicardi-Goutières Syndrome (AGS)	Mutations in genes encoding enzymes that breakdown DNA and RNA in the cytosol including TREX1, RNASEH2A, RNASEH2B, and RNASEH2C. Mutations in the SAMHD1 gene which encodes an enzyme that helps regulate the amount of available dNTPs and which plays a part in removing ribonucleotides accidentally incorporated into DNA.	TREX1, RNASEH2A, RNASEH2B, and RNASEH2C mutations: Increased L1 expression and retrotransposition and accumulation of DNA and RNA in the cytosol. SAMHD1 mutation: Increased L1 expression and retrotransposition and higher availability of dNTPs for reverse transcription in the cytosol.
Ataxia–telangiectasia (AT)	Mutations in the gene ataxia telangiectasia mutated (ATM), which encodes a serine/threonine kinase involved in DNA damage sensing and repair signaling.	Increased L1 copy number and increased length of L1 insertions before truncation.
Autism spectrum disorder (ASD)	Genetic and environmental factors: Genetic: CNVs, e.g. duplication or deletion at 16p11.2 and mutations of ASD-associated genes. Environmental: risk factors include parental age at conception, maternal nutrition, infection during pregnancy and prematurity.	Increased L1 copy number and higher levels of L1 ORF1 and ORF2 mRNA; increase in L1 activity may be due to the significant reduction in glutathione redox status in the brain and other tissues of autistic patients, as L1 retrotransposition has been associated with oxidative stress.
Schizophrenia	Genetic and environmental factors: Genetic: CNVs, e.g. deletion at 22p11.2 and mutations of schizophrenia-associated genes. Environmental: risk factors include stress, childhood trauma, parental age at conception, maternal nutrition, infection during pregnancy and prematurity.	Increase in L1 copy number; L1 insertion sites were preferentially localized to synapse- and schizophrenia-related genes.
Cocaine and methamphetamine abuse	Genetic and environmental factors: Genetic: Multiple genes are believe to predispose people to addictive behaviors Environmental: substance abuse	Higher L1 retrotransposition and increased L1 ORF1 mRNA and ORF2p levels
Frontotemporal Lobar Degeneration (FTLD) and Amyotrophic Lateral Sclerosis (ALS)	Genetic and age-related: Genetic: Mutations in various genes including Tau/MAPT, PGRN, C9ORF72, VCP, CHMP2B, TARDBP, and FUS. Age-related: Loss of neurons and build-up of TDP-43-positive cytoplasmic inclusions in ∼40% of cases; environmental factors are being investigated.	Normal silencing or regulatory action of TDP-43 on RTE expression may be lost when TDP-43 protein function is compromised, and it may be reverse transcriptase activity which results in the TDP-43 pathological toxicity observed in FTLD.
Aging	Age-related: Accumulation of DNA damage and epigenetic changes	LINEs, SINEs and LTRs become derepressed.

## Rett Syndrome

Rett Syndrome (RTT) is a rare, usually sporadic genetic postnatal neurological disorder that shows symptoms at 6–18 months of age and leads to severe impairments, including slow development, intellectual disability, seizures, and autistic behaviors. It is caused by mutation of the X-linked gene methyl CpG binding protein 2 (MECP2), which leads to abnormal epigenetic regulation (Amir et al., 1999). MeCP2p selectively binds to CpG dinucleotides in target genes and mediates transcriptional repression through interaction with histone deacetylase and the corepressor Sin3Ap (Jones et al., 1998; Nan et al., 1998).

RTT was the first neurological disorder where disease-related genetic mutations were found to result in increased levels of L1 retrotransposition in neurons and neuronal precursors (Muotri et al., 2010). MeCP2p normally targets L1 5′ UTR sequences, which results in methylation-dependent repression of the L1 promoter (Yu et al., 2001; Klose et al., 2005). In both MECP2^−/−^ mice and RTT NPCs engineered to carry an L1–EGFP construct, Muotri et al. (2010) found increased L1 retrotransposition in the brains of the transgenic mice and the RTT NPCs compared to controls. For example, there were 2.8 to 6.3-fold increases in L1 retrotransposition in the cerebellum, striatum, cortex, hippocampus, and olfactory bulb neurons in the MECP2^−/−^ mice compared to the wildtype mice. The brains of RTT patients also were analyzed for L1 ORF2 copy number by qPCR and had signiﬁcantly higher L1 ORF2 copy numbers compared to age and gender-matched controls and heart tissues from the same patients. Using chromatin immunoprecipitation (ChIP) and qPCR, Muotri et al. (2010) found higher levels of MeCP2 protein associated with endogenous L1 promoter regions in neural stem cells compared to neurons. Thus, a reduction in methylation-dependent repression is the likely mechanism for the increased levels of L1 expression and retrotransposition that are observed during neuronal differentiation (**Figures 2D–F**) (Muotri et al., 2010).

Zhao et al. (2019) also studied the impact of MeCP2 dysfunction in RTT patients on somatic L1Hs retrotransposition. They used a PCR-based targeted bulk sequencing approach to analyze 20 postmortem tissues from five RTT patients and matched healthy controls. They identified 9,181 somatic insertions in neuronal and non-brain tissues and also validated somatic L1Hs insertions in cortical neurons and non-brain tissues. RTT patients had more somatic insertions than the controls in both brain and other tissues and had a higher proportion of clonal somatic L1Hs insertions (Zhao et al., 2019). 

Further evidence of increased retrotransposition in RTT and other neurological disorders came from a recent study by Jacob-Hirsch et al. (2018). The study performed whole genome analysis of NPCs in 20 brain samples and 80 non-brain samples from normal patients and patients with several neurodevelopmental disorders [RTT, ataxia–telangiectasia, autism and tuberous sclerosis complex (TSC)]. The highest levels of L1Hs and *Alu* Y retrotransposition were observed in the NPCs from diseased brains, with lower levels of L1Hs and *Alu* Y in NPCs from non-diseased brains and non-brain tissues. Members of the L1Hs and *Alu* Y families are the youngest and most active RTEs in humans. The overall number of L1Hs insertions in these neurological diseases was 8.5 fold higher than the normal brain controls, and somatic L1Hs insertions in particular were 14.7 fold higher than controls (Jacob-Hirsch et al., 2018). In addition, the majority of somatic brain retrotransposition events (57%) had inserted into pre-existing repetitive elements. The authors hypothesized that pre-existing retrotransposons act as “lightning rods” for insertions to fine-tune gene expression while also avoiding the creation of detrimental insertions (Jacob-Hirsch et al., 2018).

## Aicardi-Goutières Syndrome (AGS)

Another instance of genetic mutations that increase the frequency of L1 retrotransposition in the human brain is exemplified by Aicardi-Goutières syndrome (AGS). AGS is a rare inherited encephalopathy resulting in severe mental and physical handicaps. About 20% of the cases are an early-onset form where infants are born with neurological and liver abnormalities including enlargement of the liver and spleen and elevated liver enzymes. Symptoms closely resemble a congenital viral infection of the brain, including elevated levels of type I interferon (IFN) in the serum and cerebrospinal fluid (Crow et al., 2014). Mutations in several genes that are involved in the degradation of DNA and RNA in the cytosol can cause AGS, including TREX1, RNASEH2A, RNASEH2B, and RNASEH2C. Mutations in the SAMHD1 gene, which encodes an enzyme that helps regulate the amount of available dNTPs to control viral infections (and removes ribonucleotides that are accidentally incorporated into DNA), also can cause AGS. Mutations in these genes results in the accumulation of DNA and RNA in the cytosol, which in turn, triggers a Type 1 IFN-induced immune response in multiple body systems (Crow and Rehwinkel, 2009; Crow et al., 2014). In the brain and spinal cord, there is damage to the myelin sheath surrounding and protecting the nerve cells; it is the loss of myelin that is responsible for the symptoms of AGS.

But how is L1 linked to AGS? Stetson et al. (2008) first observed increased L1 transcript accumulation and AGS viral infection symptoms in patients with mutations in the Three-prime Repair EXonuclease 1(TREX1) gene. They hypothesized that TREX1p may have a regulatory role in the IFN-stimulatory DNA (ISD) pathway through its ability to metabolize cytoplasmic DNA. Consistent with this hypothesis, endogenous TREX1 DNA substrates from inflamed hearts of TREX1-deﬁcient mice were increased by 32-fold compared to matched wild-type hearts. Sequencing of the extrachromosomal DNA from TREX1 KO hearts revealed that 22% of the clones were derived from retroelements compared to 7% of the wild-type clones. Likewise, 48% of the retroelement-derived DNAs in TREX1 KO hearts were from L1 retrotransposons, 40% were from LTR endogenous retroviruses, and 12% were from SINE elements. Thus, extrachromosomal RTE DNAs appear to be major substrates of TREX1p.

The finding that retroelements are TREX1 substrates was validated using two genetically marked retroelements: 1) a human L1 element and 2) an LTR-containing murine intracisternal type A particle (IAP), each containing a neomycin retrotransposition indicator cassette. In HeLa cells, transfection with the wild-type IAP plasmid resulted in abundant neomycin-resistant colonies indicating the production of retrotransposition events. However, when an expression vector for TREX1 was co-transfected, the retrotransposition efﬁciency was reduced to less than 40% of the controls. In similar experiments with L1, the co-transfected TREX1 reduced L1 retrotransposition by over 80% (Stetson et al., 2008).

An increase in extrachromosomal L1 retrotransposon DNA also was observed in TREX1-deficient neural cells and human pluripotent stem cells (Thomas et al., 2017). Two cell lines were generated using CRISPR/Cas9 to make distinct TREX1 mutations and a third line was generated by inducing pluripotency in fibroblasts from a patient who had a homozygous mutation in TREX1. The cell lines were then differentiated into NPCs, neurons, cortical organoids, and astrocytes. In the TREX1-deficient lines, the amount of extrachromosomal DNA was correlated with the level of neurotoxicity exhibited throughout the process of differentiation, and neurotoxicity was rescued in cells treated with reverse transcriptase inhibitors (RTi). Further, RTi treatment reduced indicators of IFN response [e.g. IFNβ and IFNα13 expression, pIRF3, and STING-dependent interferon-stimulated genes (ISGs)] in TREX1 deﬁcient astrocytes to levels similar to the controls. Presumably, the RTi’s were inhibiting the L1 reverse transcriptase, thereby reducing the amount of extrachromosomal L1 DNA that was generated in the cytosol (although how and why L1 DNA is in the cytosol is a mystery).

Sequencing of extrachromosomal DNA from one of the TREX1-deﬁcient NPC lines, its isogenic control, and an RTi-treated TREX1-deﬁcient line, showed that extrachromosomal L1 DNA was abundant when TREX1 was inactive, and in particular, there was 70% more L1Hs in the TREX1- deﬁcient line compared with the control. In contrast, the RTi-treated, TREX1-deficient cell line had nearly normal levels of L1 and L1Hs extrachromosomal DNAs. This is consistent with the extrachromosomal DNAs being generated by the L1 RT (encoded by L1 ORF2p). Interestingly, later work by Li et al. (2017) showed that TREX1-mediated L1 suppression is not dependent on TREX1 exonuclease activity. Using multiple TREX1 mutants that were defective in digesting DNA, they showed there was still a similar potency against L1 when compared to wild-type TREX1.

The most common cause of AGS, found in over 50% of AGS patients, is biallelic mutation of one of the three RNase H2 subunits (RNase H2A, RNase H2B, and RNase H2C) (Crow and Manel, 2015). Such mutations also lead to the accumulation of cytosolic L1 DNA, indicating another possible link between AGS and L1. Pokatayev et al. (2016) introduced an RNase H2 AGS mutation into the mouse RNASEH2A gene, resulting in homozygous RNASEH2A-G37S/G37S (G37S) mice. The analogous mutation causes severe early onset AGS in humans. The mouse mutant had reduced RNase activity and increased levels of cytosolic L1 DNA (Pokatayev et al., 2016).

Benitez-Guijarro et al. (2018) later found that L1 retrotransposition was severely compromised in three diverse cell lines that had RNase H2 null mutations introduced by CRISPR/Cas9 genome editing (HeLa, HCT116, and U2OS). Likewise, overexpression of the three RNase H2 subunits in HeLa and U2OS cells resulted in significant increases in retrotransposition compared to controls, suggesting that these factors facilitate L1 retrotransposition. Based on these data, Benitez-Guijarro et al. (2018) proposed that RNase H2 degrades the L1 RNA after reverse transcription, allowing retrotransposition to be efficiently completed. Their model can help to explain how L1 can function without its own active RNase H domain. Their findings provide further support for the idea that the by-products of active retrotransposition drive AGS pathology.

Mutation of the SAMHD1 gene also causes AGS. In work by Zhao et al. (2013), the SAMHD1 enzyme was shown to be a strong modulator of L1 retrotransposition. SAMHD1-expressing plasmids transfected into HEK293T and HeLa cells caused a potent and dose-dependent suppression of L1 activity in a plasmid-based assay for retrotransposition. Likewise, small interfering RNAs (siRNAs) that targeted endogenous SAMHD1 and reduced its activity in HEK293T cells produced a 230% increase in L1 retrotransposition. Debilitating point mutations in SAMHD1 that have been identiﬁed in AGS patients likewise led to increased levels of L1 retrotransposition in several mammalian species. When SAMHD1 was overexpressed in HEK293T cells, L1 ribonucleoprotein particles (L1-RNPs) that were isolated from these cells supported reduced levels of ORF2p-mediated endogenous reverse transcription of L1 mRNA. Likewise, ORF2p expression was reduced by 62% in these L1-RNPs. A similar inhibition of ORF2p-mediated *Alu* and SVA retrotransposition also was observed (Zhao et al., 2013).

Despite the evidence that SAMHD1 is a modulator of L1, Upton et al. did not observe an increase in L1 mobilization in the hippocampus of a SAMHD1-deﬁcient AGS patient (Upton et al., 2015). They used bulk RC-seq and single cell genomics on a post-mortem hippocampus and ﬁbroblasts from an AGS patient carrying two loss-of-function SAMHD1 mutations. By analyzing 21 neuronal nuclei that they isolated from the hippocampus of this patient, they identified 373 putative somatic L1 insertions and an estimated 8.0 insertions per neuron. However, they estimated that there were 13.7 somatic L1 insertions per neuron in control hippocampal neurons, and the difference was even more significant when using age- and gender-matched control hippocampal neurons. A signiﬁcantly lower L1 copy number also was observed by qPCR in the AGS patient’s hippocampus versus controls and these data were strongly correlated with the mean somatic L1 insertion frequencies estimated by single-cell RC-seq. The data led them to conclude that increased L1 mobilization in this patient’s hippocampus was unlikely (Upton et al., 2015). It is unclear why this patient did not have higher levels of L1 retrotransposition in the absence of repressive SAMHD1 activity. Given that only a single patient was examined, additional patients may be needed to reconcile these studies with those outlined above in cultured cells.

## Ataxia–Telangiectasia (AT)

A third disorder where genetic mutations increase the frequency of L1 retrotransposition in neurons is found with ataxia–telangiectasia (AT). AT is a rare inherited neurodegenerative disorder that is caused by mutations in the ataxia telangiectasia mutated (ATM) gene, which encodes a serine/threonine kinase that is involved in DNA damage sensing and repair signaling. AT mainly affects the immune and nervous systems and is characterized by progressive difficulty with coordinating movements beginning in early childhood (Rothblum-Oviatt et al., 2016).

In a study of AT by Coufal et al. (2011), an increase in neuronal L1 retrotransposition was found in a variety of experimental settings. For example, ATM KO mice carrying an L1–EGFP transgene had increased levels of L1 retrotransposition in brain samples, but not in other somatic tissues, and the largest increase was seen in the hippocampus. In hESC-derived NPCs expressing ATM shRNA, a twofold increase in L1 retrotransposition was observed in a plasmid-based retrotransposition assay. These results also were confirmed in humans: hippocampal sections of postmortem human brain tissue from seven AT patients and seven controls were analyzed for L1Hs copy number using qPCR. An increase in L1 ORF2 copy number was found in AT neurons relative to age- and sex-matched controls.

Coufal et al. (2011) also investigated the mechanism for enhanced L1 retrotransposition in ATM-deﬁcient cells. Because the L1 ORF2p RT has been shown to be highly processive *in vitro* and yet most genomic L1 insertions are 5′ truncated, Coufal et al. (2011) hypothesized that cellular DNA repair and damage sensing proteins may play a role in truncating L1 insertions. Using LRE3–EGFP constructs containing either one or two 500-bp spacer regions between the LRE3 polyadenylation site and the EGFP indicator, the fraction of EGFP positive cells was found to be reduced with increasing insert length in the control cells, whereas there was no change (or less change) with increasing insert lengths in the ATM deficient cells. Coufal et al. (2011) proposed that, during L1 integration, retrotransposition intermediates may be recognized by ATM as DNA damage, which in turn would stall replication and cause 5′ truncation of the retrotransposition event.

Additional supporting evidence of increased L1 activity in AT patients was discussed earlier where Jacob-Hirsch et al. (2018) used postmortem brain samples from patients with AT, as well as RTT, autism and TSC, and showed that L1Hs retrotransposition events were increased in the brains of these patients compared to controls; the overall levels of L1Hs insertions were 8.5 fold higher in the four neurodevelopmental disorders vs. normal controls, and the somatic L1Hs insertions specifically were 14.7 fold higher than controls. These results are in good agreement with those from the Coufal study (Coufal et al., 2011).

As with the other two examples where disease-related genetic mutations led to increased frequencies of neuronal L1 expression and retrotransposition (i.e., Rett and AGS), L1 activity likely is not the underlying cause of AT. However, it is possible that it contributes to some of the symptoms or to the progression of these diseases. In addition to the potentially deleterious effects that might be caused by somatic retrotransposon insertions (particularly those that disrupt genes), the endonuclease of L1 ORF2p has the potential to cause DNA damage even in the absence of a successful retrotransposition event (Kines et al., 2014; Christian et al., 2016). Likewise, DNA-mediated damage at integration sites appears to be targeted by ATM, and additional DNA damage/repair pathways also may send somatic cells towards apoptosis, necrosis, or senescence. As outlined above, another possible mechanism is that the immune system may be activated by the by-products of retrotransposition (ORF1p, ORF2p, L1 RNA, and L1 DNA). Additional work is needed to more fully assess the potential impact of RTE on the progression of these diseases.

## Autism Spectrum Disorder (ASD)

Unlike RTT, AGS, and AT, a single gene (or small group of genes) has not been found to cause autism spectrum disorder (ASD). However, estimates of the heritability of ASD from twin studies are as high as 93% indicating that genetic factors contribute to the disease (Ronald and Hoekstra, 2011). The Centers for Disease Control and Prevention (CDC) reports that, if one identical twin has autism, the other will be affected 36 to 95% of the time and if one non-identical twin has autism, the other twin will be affected about 31% of the time (Ronald et al., 2006; Taniai et al., 2008; Rosenberg et al., 2009; Hallmayer et al., 2011). The lack of complete concordance in ASD studies on identical twins suggests that the disorder may be due to a combination of genetic and environmental factors (Karimi et al., 2017). There are now over 700 genes and over 2,000 copy number variations (CNVs) with potential links to ASD in a professionally curated database for the autism research community called SFARI Gene (Abrahams et al., 2013). According to the CDC, approximately 1 in 59 children is diagnosed with ASD, with a rate of 1 in 37 boys versus 1 in 151 girls (Baio et al., 2018). Autism is characterized by issues with verbal and nonverbal communication, social skills and repetitive behaviors. ASD can be diagnosed as early as age 2, although most children are not diagnosed until after age 4 (Baio et al., 2018). Of those affected by ASD 31% have an intelligence quotient (IQ) < 70, 25% are in the borderline range of 71–85, and 44% are in the average to above average range with an IQ >85 (Baio et al., 2018).

Studies of genetic changes associated with ASD have mainly been focused on germline changes, however recent studies have shown increased levels of somatic L1 activity in postmortem brains of ASD patients (Jacob-Hirsch et al., 2018; Shpyleva et al., 2018). As discussed earlier, Jacob-Hirsch et al. (2018) showed that in autism, as well as RTT, AT, and TSC, L1Hs retrotransposition events were increased in the brains of patients with these neurodevelopmental diseases. L1Hs insertions were 8.5 fold higher in the four neurodevelopmental disorders vs. normal controls, and somatic L1Hs insertions in particular were 14.7 fold higher (Jacob-Hirsch et al., 2018). Therefore, the increases in somatic L1 activity observed in these patients might also impact ASD through inflammation or some other mechanism.

In postmortem brain samples from 13 individuals with ASD, Shpyleva et al. (2018) found a signiﬁcant increase in both L1 ORF1 and ORF2 mRNA in the cerebellum, but not in the three other brain regions analyzed (frontal cortex, anterior cingulate, and auditory processing). However, they found no signiﬁcant differences in L1 copy numbers in the cerebellum or the other three regions. Using ChIP to measure MeCP2p binding to the L1 5′ promoter region, a negative correlation between MeCP2p binding to L1 5′ UTR and ORF1 expression was found in autism brains, but not in the controls, leading them to propose that reduced MeCP2p binding contributes to the increase in L1 ORF1 expression seen in the autism cerebellum (Shpyleva et al., 2018). The group found no differences in DNA methylation density in L1 5′ UTR, ORF1, and ORF2 sequences between autism and control samples. However, using ChIP they found that trimethylation of histone H3K9 (H3K9me3), which is responsible for the formation of condensed heterochromatin and prevents L1 activation, was significantly reduced at L1 ORF1 and ORF2 sequences but not at the 5′ UTR in the autism samples (Shpyleva et al., 2018).

Because Shpyleva et al. (2018), and others previously, had reported a significant reduction in glutathione redox status, i.e. the ratio of active reduced to inactive oxidized glutathione disulfide (GSH/GSSG), in the brain and other tissues of autistic patients, and because L1 retrotransposition has been associated with oxidative stress (Giorgi et al., 2011), they looked at the GSH/GSSG redox status and L1 ORF1 and ORF2 expression. They found that increased L1 expression and redox stress were significantly correlated with the autism cerebellar samples but not the matched controls. While this study was specific to autism, increased L1 expression induced by redox imbalance and oxidative stress may be a factor in other neurological disorders as well (Nunomura et al., 2001; Giorgi et al., 2011; Trivedi et al., 2014).

## Schizophrenia

According to the National Institute of Mental Health, schizophrenia is estimated to affect between 0.25 and 0.64% (Kessler et al., 2005; Wu et al., 2006) of individuals in the U.S. with symptoms starting in late adolescence or early adulthood, although unusual behaviors are sometimes observed in childhood (McGrath et al., 2008). Whereas schizophrenia sometimes runs in families perhaps indicating a genetic component, environmental factors, particularly in early life, such as stress and exposure to viruses or poor nutrition before birth, also are thought to contribute to the disease. As reviewed by Van et al. (2017), the strongest known genetic risk factor for schizophrenia is the 22q11.2 deletion, with 0.5–1% of schizophrenics having the 22q11.2 deletion and one in four individuals born with this deletion developing schizophrenia. The deletion occurs due to a non-allelic meiotic recombination during spermatogenesis or oogenesis (McDonald-McGinn et al., 2015). CNVs overall account for an estimated 3.5–5% of schizophrenia (Van et al., 2017). The 22q11.2 deletion is the most common CNV found in individuals with schizophrenia, but schizophrenia is another disease where no single gene (or small group of genes) has been found to be the cause.

Bundo et al. (2014) found that L1 ORF2 copy numbers were increased in neurons from the postmortem prefrontal cortexes of patients with schizophrenia. In addition, increased L1 copy number was found in induced pluripotent stem cell-derived neurons containing a 22q11 deletion. Whole genome sequencing showed that the L1 insertion sites were preferentially localized to synapse- and schizophrenia-related genes. The authors proposed that the environmental and/or genetic risk factors that increased L1 retrotransposition in neurons may be contributing factors to the pathophysiology of the disease (Bundo et al., 2014). Further confirmation of L1 insertions in genes implicated in schizophrenia came from a later study by Doyle et al. (2017). Genomic sequences were amplified from the flanking 3′-side of L1s in neurons from the postmortem prefrontal cortexes of 36 schizophrenia patients. A significant increase in L1 insertions into genes implicated in schizophrenia was observed, although the L1 insertions were mainly germline insertions, with only one being a possible somatic insertion generated during early development (Doyle et al., 2017).

Immune activation models simulating both viral infection and inflammation also have been used to investigate possible links between perinatal environmental risk factors for schizophrenia and L1 activity. Viral infection was simulated by injecting pregnant mice with polyinosinic:polycytidylic acid [poly(I:C)], which mimics viral double-stranded RNA. Inflammation was simulated by chronic injection of epidermal growth factor (EGF) into neonatal macaques. In both the mouse and macaque models, an increase in brain L1 copy number was observed in response to these two perturbations, indicating that the L1 content of the brain likely is influenced by early environmental factors (Bundo et al., 2014).

One of the environmental risk factors for schizophrenia is early childhood trauma, and the association of this risk factor with L1 expression was demonstrated by Misiak et al. (2015). Here, the authors looked at L1 methylation in peripheral blood leukocytes from 48 patients with ﬁrst-episode schizophrenia and 48 healthy controls. In the patients with a history of childhood trauma they found signiﬁcantly lower L1 methylation levels relative to the patients without childhood trauma or healthy controls (Misiak et al., 2015). As discussed by Misiak et al. (2015), patterns of DNA methylation in peripheral tissues of schizophrenia patients have been shown to be indicative of that in the brain of the same patient, suggesting that the epigenetic dysregulation observed in blood tissues may be systemic. Further confirmation of epigenetic dysregulation of L1 in schizophrenia came from recent work by Li et al. (2018), who looked at L1 methylation of peripheral blood samples from a cohort of Han Chinese including 92 schizophrenia patients, 99 bipolar disorder patients, and 92 controls. Li et al. (2018) also found hypomethylation of L1 in the schizophrenia patients as well as those with bipolar disorder. Additional work will be needed to determine whether these L1 methylation results with peripheral blood samples are truly indicative of L1 methylation in the brain.

## Cocaine and Methamphetamine Drug Abuse

The first study to find an impact of substance abuse on L1 expression was by Maze et al. (2011) investigating the impact of cocaine exposure on the nucleus accumbens (NAc), an area of the brain with a central role in the reward circuit. Mice were exposed to both acute (one dose) or repeated doses (seven daily doses) of cocaine, which led to dynamic ﬂuctuations in H3K9me3 (Maze et al., 2011). The H3K9me3 mark is thought to primarily reside in silenced, noncoding regions of the genome. With repeated cocaine exposure, a persistent decrease in repressive methylation in the NAc was found. Using ChIP-Seq to examine H3K9me3 binding in the NAc, H3K9me3 was found predominately in intergenic regions and repeated cocaine exposure decreased H3K9me3 abundance at several speciﬁc retrotransposons (LINE-1, SINE, and LTRs). Using qRT-PCR, a signiﬁcant increase in expression for one of the L1 sites was confirmed (Maze et al., 2011). Thus, chronic drug exposure was shown to result in de-repression of retrotransposons (mainly L1), and long-lasting changes in gene expression that may contribute to the pathophysiology of drug addiction.

An ex vivo study by Doyle et al. (2017) examined L1 insertions in neurons isolated from the postmortem medial prefrontal cortexes of 30 cocaine addicts who died of cocaine overdose. Most of the novel L1 insertions identified were likely germline or developmental *de novo* mutations; a small fraction of the insertions were possibly somatic L1 insertions that were associated with cocaine addiction. No changes in L1 transcription or retrotransposition were observed in bulk medial prefrontal cortex tissues from individuals with cocaine addiction. However, the novel L1s in these individuals were enriched in genes that previously have been associated with cocaine addiction. This supported the authors’ hypothesis that inherited or somatic L1s in neuronal genomes may predispose an individual to cocaine addiction (Doyle et al., 2017).

*In vitro*, a study by Okudaira et al. (2014) found that cocaine and methamphetamine (METH) induced L1 retrotransposition in cultured SH-SY5Y neuroblast cells, and cultured PC12 rat pheochromocytoma cells, but did not induce L1 retrotransposition in cultured HT1080 human fibrosarcoma cells nor HeLa cells. The affect could be inhibited with reverse transcriptase inhibitors, suggesting that the cocaine and METH induction of L1 was dependent on the L1 RT. Further, DSBs were ruled out as the stimulus for retrotransposition, as the authors did not find an increase in the expression of γ-H2AX by Western blot and did not find focus formation of γ-H2AX by immunohistochemistry (IHC) (Farkash et al., 2006). Using RNA interference experiments combined with add-back of siRNA-resistant cDNAs, they determined that both cocaine and METH stimulated retrotransposition by promoting the recruitment of L1 ORF1 to chromatin in a cAMP response element-binding protein (CREB)-dependent manner (Okudaira et al., 2014).

In contrast to the previous study, which examined non-toxic doses of METH, Moszczynska et al. (2015) examined the effect of neurotoxic METH doses on L1 expression in the striatum and hippocampus of the adult rat brain. An increase in L1 activity, measured by L1 ORF1 mRNA and ORF2p levels, was found in the subgranular zone, within the dentate gyrus and the subventricular zone. In addition, an increase in L1 ORF1 copy number was found in the striatum, hippocampus, and liver, but not in muscle tissue. Overall, this study confirmed that METH treatment stimulates L1 retrotransposition in both neuronal and non-neuronal cells (Moszczynska et al., 2015).

## Frontotemporal Lobar Degeneration (FTLD) and Amyotrophic Lateral Sclerosis (ALS)

Unlike the disorders discussed so far, which show symptoms relatively rapidly, frontotemporal lobar degeneration (FTLD) and cognitive decline are typically associated with aging, or from a cumulative effect of perturbations or alterations in cellular mechanisms over time. FTLD is a process of shrinkage of specific areas of the brain that regulate behavior, personality, and language. Up to 40% of patients with this disease have a family history of FLTD. FTLD is the second most common cause of dementia in patients younger than 65 years of age and it accounts for another 25% of dementia cases in patients older than 65 years (Young et al., 2018). The accumulation of a protein called transactive response DNA binding protein 43 kDa (TDP-43) in cytoplasmic inclusions is a pathological characteristic of ∼40% of patients with FTLD. Such inclusions also are found in patients with amyotrophic lateral sclerosis (ALS), Alzheimer’s disease, and other neurodegenerative disorders (Cohen et al., 2011). The aggregation-prone TDP-43 is a binding protein of both single stranded DNA and RNA and it has multiple functions in transcriptional repression, pre-mRNA splicing, and translational regulation.

Based on data from a meta-analysis of publicly available TDP-43 data, protein:RNA target binding datasets, and mRNA expression datasets, Li et al. (2012) hypothesized that RTE over-expression may contribute to TDP-43-mediated neurodegeneration. Using deep sequencing datasets of RNA targets that co-purify with immunoprecipitated mouse, rat, or human TDP-43, and data for gene expression changes after knockdown or over-expression of TDP-43 in mice, Li et al. found that RTE-derived transcripts are targets of TDP-43 (including many SINEs, LINEs and LTR transcripts). Based on these results, Li et al. suggested that the normal silencing or regulatory action of TDP-43 on RTE expression is lost when TDP-43 protein function is compromised, and that the accumulation of RTE-derived RNAs or proteins may contribute to TDP-43-mediated neurodegenerative disorders by causing a DNA-damage stress response or toxic effects (Li et al., 2012).

The work above was continued *in vivo*, in a *Drosophila* model of FTLD where human TDP-43 (hTDP-43) was overexpressed from a hTDP-43 transgene (Krug et al., 2017). This, in turn, led to a concentration-dependent phase separation of TDP-43p to form liquid droplets that drove fibrilization, with aggregation of TDP-43p in cytoplasmic inclusions, clearance of TDP-43p from the nuclear compartment, and neurological defects. In *Drosophila* neurons and glia expressing human hTDP-43p, toxic cytoplasmic accumulation of TDP-43p was associated with an increase in the expression of transposon RNAs (mostly retrotransposons). An LTR retrotransposon, i.e. gypsy, was selected to test the impact of hTDP-43p on induced retrotransposon expression. In glia, gypsy was induced by hTDP-43p expression and the resulting toxicity could be partially rescued through the use of an RTi, suggesting that gypsy caused the degenerative phenotypes in *Drosophila*. Further, the shortened lifespan in the hTDP-43p-expressing flies was fully rescued by blocking the expression of Loki expression, showing that hTDP-43p toxicity is predominately caused by Loki/Chk2 activation of DNA damage-mediated programmed cell death (Krug et al., 2017). This study suggests that TE activity, and in particular RT activity, is responsible for the pathological toxicity observed in this *Drosophila* model of FTLD.

The same group used this *in vivo* model to study the mechanisms mediating cell-to-cell spread of toxicity seen in frontal temporal dementia (FTD—a subtype of FTLD) and ALS (Chang and Dubnau, 2019). Using the hTDP-43p-expressing *Drosophila* model, Chang et al. initiated toxic expression of human TDP-43 within small groups of glial cells. Interestingly, the focal onset of TDP-43 toxicity was found to kill adjacent neurons in a manner that mimicked FTD and ALS. DNA damage foci and apoptotic signaling also were found in progressively larger numbers of neurons associated with gypsy replication. On the basis of these results in the *Drosophila* model, the group proposed that human retroviruses such as HERV-K might contribute to TDP-43-mediated neurodegeneration in humans with FTD and ALS (Chang and Dubnau, 2019).

The Hammell lab recently analyzed the transcriptomes of 148 ALS cortex tissue samples and identified three ALS clusters based on the observed gene expression profiles: (1) a major cluster of samples with oxidative and proteotoxic stress (61% of patients), (2) a second cluster with glial activation and inflammation (19% of patients) and (3) a third cluster with retrotransposition reactivation (20% of patients) (Tam et al., 2019). Since oxidative stress and inflammation previously have been implicated in ALS, the investigators focused on the retrotransposon cluster, which included elements from the LINE, SINE, and LTR classes. They sequenced the RNAs that were bound to TDP-43 protein in human SH-SY5Y neuroblastoma cells using an enhanced cross-linking and immunoprecipitation protocol (eCLIP-seq) (Van Nostrand et al., 2016), and found that transposable elements accounted for 31% of all mapped peaks. Their data support a model whereby human transposons are normally silenced by TDP-43, but that such silencing is reversed in a subset of ALS patients, which may lead to cellular toxicity (Tam et al., 2019).

In a separate study Liu et al. showed that TDP-43 normally inhibits L1 retrotransposition (Liu et al., 2019). In particular, they used subcellular fractionation and FACS to isolate diseased neuronal nuclei with depleted TDP-43 (TDPneg nuclei) from the post-mortem brain samples of frontotemporal degeneration-amyotrophic lateral sclerosis (FTD–ALS) patients (Liu et al., 2019). They found that loss of TDP-43 was associated with chromatin de-condensation around L1 elements and increased L1 content. Using HeLa cells transfected with a retrotransposition expressed L1–GFP plasmid and a plasmid encoding Cas9 with one of two different guide RNAs to knock out TDP-43 expression, they found a significant increase in L1 retrotransposition in the absence of TDP-43. These data provide additional support that TDP-43 normally silences transposons. Based on this work, the authors suggested that pharmacologic inhibition of retrotransposition activities using antiretroviral drugs might be a way to mitigate the neurotoxic effects of TDP-43 pathology in FTD-ALS patients (Liu et al., 2019).

## Aging

Epigenetic changes are known to occur with aging. For example, Fraga et al. (2005) showed that during early life, monozygous twins are epigenetically indistinguishable; however older twins exhibit significant differences both in DNA methylation levels and the genomic distributions of methylated sites. Recently, multiple studies have shown that the loss of epigenetic silencing with aging reactivates RTEs and this may be a contributing factor to age-related mental decline and diseases. A longitudinal study of elderly individuals in Massachusetts examined the level of DNA methylation by qPCR pyrosequencing over 8 years. Based on 1,097 blood DNA samples from 718 elderly subjects between 55–92 years of age, a gradual linear decline with age was found in *Alu* methylation but not L1 methylation (Bollati et al., 2009).

Research also has shown that LINEs, SINEs, and LTRs become de-repressed and start actively transposing during replicative senescence *in vitro*. Using formaldehyde assisted isolation of regulatory elements (FAIRE) to map genome-wide chromatin conformations in human fibroblasts in cell culture, chromatin was found to undergo extensive changes during replicative senescence with an overall closing of chromatin in euchromatic gene-rich regions and a relaxation of heterochromatin in gene poor and pericentromeric regions (De Cecco et al., 2013). With respect to retrotransposons in senescent cells, the chromatin of L1, *Alu*, and SVA was more open, L1 and *Alu* elements had higher levels of expression, and L1 copy number was increased, compared to controls. These changes were confirmed in mouse liver samples from 5, 24, and 36 month old mice. Expression of both LINEs and SINEs in liver and skeletal muscle tissues was found during normal aging, with increased copy numbers at an advanced age. Further, environmental factors were observed to influence this process as caloric restriction, which slows down aging, was found to counteract the changes in RNA levels of the retrotransposable elements (De Cecco et al., 2013).

In *Drosophila* melanogaster, two LINE-like transposable elements (R1 and R2), and an LTR retrotransposon (gypsy), were found to be highly active in the *Drosophila* brain during normal aging (Li et al., 2013). By mutating an endogenous repressor of transposons and genes, i.e., *Drosophila* Argonaute 2 (dAgo2), there was increased R2 and gypsy expression in the brain of young *Drosophila* and the increased expression was correlated with an accelerated age-dependent memory impairment and shortened lifespan. In addition, there was a significant delay in mortality and age-dependent memory impairment when an RNAi transgene was used to target Loki/Chk2. Based on these results, the authors suggested that transposon activation may be a contributing factor in age-dependent loss of neuronal function (Li et al., 2013). Recently Chang et al. (2019) confirmed increased gypsy replication with both aging and mutation of dAgo2.

These studies have shown that a relaxing of RTE silencing occurs with age, and the aberrant expression and increased retrotransposition activity of RTEs may result in DNA damage. In total, these studies show that retrotransposons are progressively reanimated as organisms age, but the extent to which this is a symptom of or a cause of cognitive decline is unclear. Additional work is needed to determine whether the increased RTE activity that occurs over time impacts aging or is instead a symptom of aging.

## Conclusion

RTEs have actively proliferated over the past 80 million years of primate evolution. As an evolutionary response to their potentially detrimental effects on the human genome and transcriptome, silencing mechanisms have evolved to restrict RTE expression and retrotransposition. The brain is the only known somatic tissue where RTEs are de-repressed throughout the life of a healthy human. In the diseases outlined above, RTEs are improperly regulated and often are overexpressed beyond normal levels in the diseased state. Whether this is a marker of the disease or a driver is, in most cases, poorly understood. However, increased mutagenesis by mobile elements likely is detrimental, and may very well contribute to disease progression. In addition to deleterious effects from somatic retrotransposon insertions, which can disrupt genes or otherwise dysregulate gene expression, the by-products of the retrotransposition process also may have detrimental effects. For example, the ORF2p product of L1 can cause mutations and instability through its endonuclease activity and L1 expression may result in a buildup of RNA and/or DNA in the cytosol trigging an immune response, inflammation and neuron degeneration. While research shows increased levels of RTE activity in many neurological disorders, additional work is needed to establish the extent to which RTE activity impacts these disorders. Thus, a better understanding of the role of RTEs in neuronal tissues likely is an important part of understanding, preventing, and treating these disorders.

## Author Contributions

DT and SD reviewed the literature and prepared the manuscript. DT designed the figures and **Table 1**.

## Funding

This work was supported by grant R01HG002898 from the National Human Genome Research Institute, National Institutes of Health.

## Conflict of Interest

The authors declare that the research was conducted in the absence of any commercial or financial relationships that could be construed as a potential conflict of interest.
